# P-1729. Implementation of a Vancomycin AUC and Trough-based Dosing Policy Across a Large Health-system Reduced Trough Levels, Acute Kidney Injury Events, and Hospital Length of Stay

**DOI:** 10.1093/ofid/ofae631.1893

**Published:** 2025-01-29

**Authors:** Alyssa Christensen, Zachary Nelson, Rebecca Peglow

**Affiliations:** HealthPartners, Minneapolis, Minnesota; HealthPartners, Minneapolis, Minnesota; HealthPartners, Minneapolis, Minnesota

## Abstract

**Background:**

Vancomycin dosing guidelines recommend area-under-the-cure (AUC) over trough-based dosing for severe MRSA infections. Optimal dosing of non-severe or non-MRSA is unknown. Conflicting data exists on the real-world benefits of implementing AUC-based dosing methods.

**Figure:**
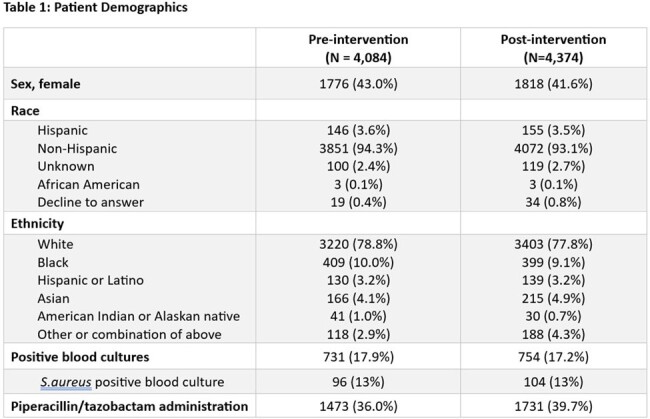

**Methods:**

A standardized vancomycin dosing policy was implemented across an 8-hospital healthsystem in MN and WI. Prior to implementation, most hospitals targeted troughs between 15-20 for severe MRSA infections. The new policy recommended AUC-based dosing for patients with known or suspected severe MRSA infections and lower trough goals (10-15) for all other patients. DoseMeRx was used to calculate AUC and adjust doses. Vancomycin levels, AKI-coded diagnoses, and length of hospital stay (LOS) were compared 6 months pre and post intervention. An approximate and conservative $7,000 cost avoidance was assigned per AKI event based on a literature review.

**Figure:**
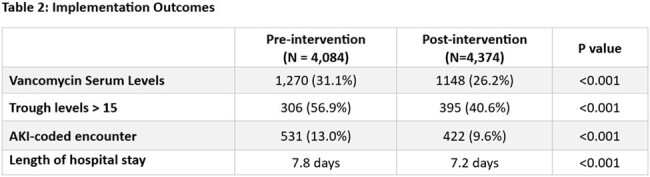

**Results:**

8,458 patient encounters were included over the 12 month study period (4084 pre, 4,374 post) representing 7,142 patients (3,517 pre, 3,825 post). Average cumulative vancomycin doses were lower in the post group by 401 mg (4,838mg vs 4,435mg). The number of trough levels greater than 15 was significantly reduced post-intervention (56.9% vs 40.6%, p < 0.001). There was a 25% relative reduction in AKI-coded encounters (13.0% vs 9.6%, p < 0.001) and reduction in average LOS (7.8 vs 7.2 days; p < 0.001 ). The number of encounters with S.aureus positive blood cultures was the same between groups (13.0%). The number of patients who received at least one dose of piperacillin/tazobactam was higher in the post-group (36.0% vs 39.7%). The reduction in AKI events was associated with a $1,029,000 cost avoidance.

**Conclusion:**

A system vancomycin dosing policy that incorporated both AUC and trough-based dosing methods was associated with a significant reduction in trough levels > 15, AKI events, and LOS. This represented a significant cost-avoidance of approximately $1,029,000 over a 6-month implementation period.

**Disclosures:**

**All Authors**: No reported disclosures

